# Nanomaterials for Biosensing Lipopolysaccharide

**DOI:** 10.3390/bios10010002

**Published:** 2019-12-21

**Authors:** Palak Sondhi, Md Helal Uddin Maruf, Keith J. Stine

**Affiliations:** Department of Chemistry and Biochemistry, University of Missouri – Saint Louis, Saint Louis, MO 63121, USA; ps2f7@mail.umsl.edu (P.S.); mm96f@mail.umsl.edu (M.H.U.M.)

**Keywords:** lipopolysaccharides (LPS), biosensing, nanomaterials, endotoxin

## Abstract

Lipopolysaccharides (LPS) are endotoxins, hazardous and toxic inflammatory stimulators released from the outer membrane of Gram-negative bacteria, and are the major cause of septic shock giving rise to millions of fatal illnesses worldwide. There is an urgent need to identify and detect these molecules selectively and rapidly. Pathogen detection has been done by traditional as well as biosensor-based methods. Nanomaterial based biosensors can assist in achieving these goals and have tremendous potential. The biosensing techniques developed are low-cost, easy to operate, and give a fast response. Due to extremely small size, large surface area, and scope for surface modification, nanomaterials have been used to target various biomolecules, including LPS. The sensing mechanism can be quite complex and involves the transformation of chemical interactions into amplified physical signals. Many different sorts of nanomaterials such as metal nanomaterials, magnetic nanomaterials, quantum dots, and others have been used for biosensing of LPS and have shown attractive results. This review considers the recent developments in the application of nanomaterials in sensing of LPS with emphasis given mainly to electrochemical and optical sensing.

## 1. Introduction

Bacterial infection has serious consequences in the human body and can be subdivided as arising from Gram-positive bacteria or Gram-negative bacteria. Amongst the many Gram-negative bacteria are infamous infectious agents for major diseases such as meningitis, pneumonia, diarrhea, and plague. While small amounts of lipopolysaccharide (LPS) released from pathogens may induce a vigorous natural response that is vital for the immune system activity against infection, the accumulation of bacterial (LPS) into the blood can cause lethal septic shock syndrome. For the most pathogenic Gram-negative bacteria, LPS transport proteins are critical and are mostly protected within the dual membrane structure.

**Figure 1:**
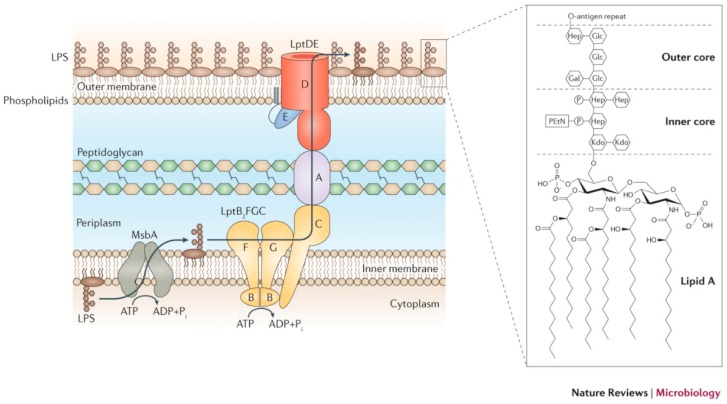
Lipopolysaccharide (LPS) transport pathway in *Escherichia coli*. LPS is synthesized on the cytoplasmic side of the inner membrane (IM) and flipped to the periplasmic side by the ATP-binding cassette (ABC) transporter MsbA. LPS is then transported to the cell surface by the LPS transport (Lpt) pathway. This pathway consists of seven essential proteins, LptA, LptB, LptC, LptD, LptE, LptF, and LptG. LPS is extracted from the IM in an ATP-dependent manner by the ABC transporter LptB2FG and transferred to LptC, which forms a complex with LptB2FG. LptC consists of a single membrane-spanning domain and a large periplasmic domain, which forms a periplasmic bridge with the soluble protein LptA and the amino-terminal region of LptD. LPS transverses the aqueous periplasmic space through this protein bridge and reaches the cell surface with the aid of the carboxy-terminal domain of LptD, which forms a β-barrel structure that is plugged by the outer membrane (OM) lipoprotein LptE. LPS is composed of lipid A, the inner and outer core oligosaccharides, and the O antigen, which is highly variable and absent in *Escherichia coli* K-12. The letters (A–G) in the figure correspond to the respective Lpt protein in the transport pathway. EtN, ethanolamine; Gal, d-galactose; Glc, d-glucose; Hep, l-glycero-d-*manno*-heptose; Kdo, 3-deoxy-d-*manno*-octulosonic acid; P, phosphate; Pi, inorganic phosphate. (Reproduced with permission from reference [2]).

Gram-negative bacteria consists of a asymmetric outer membrane, in which the inner cytoplasmic membrane wall is rich in phospholipids and the outer leaflet is comprised largely of LPS that covers 75% of the surface of the bacteria. The periplasmic space separates the outer membrane from the inner membrane. LPS is a colossal glycolipid that presents a large number of sugars on the O-antigen component into the external environment. LPS consists of three structural components: lipid A, the core oligosaccharide, and the O-antigen. A protein complex containing LPS transport protein (Lpt) creates a bridge from the inner to outer membrane and serves to accelerate transport of completed LPS to the cell surface after its synthesis in the cytoplasm [1]. The ATP-binding cassette transporter MsbA flips LPS from the cytoplasmic side to the periplasmic side of the inner membrane. The LPS transport pathway consisting of seven proteins then transports LPS to the outer membrane and the bacterial cell surface. A bridge of protein complexes is involved in helping LPS cross the periplasm to the outer membrane. A beta-barrel membrane protein provides a mechanism for LPS to be transported to the outer leaflet of the outer membrane [2]. The LPS structure and the components of the transport system are shown in Figure 1. It is known that the protein bridge must be open to the movement of the LPS which first enters the gate region without the need for ATP but then requires ATP to enter the bridge region. The transport system is proposed as unidirectional based on structural studies of the LPS transport proteins and binding assays. The seven LPS transport proteins (LptA–LptG) form a protein envelope of complexes to transfer LPS from the inner to outer membrane. The development of new antibiotics targeting LPS transport is an important goal and a peptidomimetic targeting LptD in a mouse model of sepsis so as to inhibit LPS transport successfully showed antibacterial activity [3]. LPS is crucial for the survival of bacteria as it safeguards the bacteria from the external harsh environment and toxic moieties, as well as from antibiotics. Therefore, investigation of LPS is fundamental to better understand interactions during infection and also establishing that sensing and isolation of bacteria is feasible by taking advantage of LPS–lectin interactions [4,5].

Recognition of LPS from various bacteria can be monitored using LPS receptors as well as accessory proteins. CD14 (cluster of differentiation 14) plays an important role accompanying the toll-like receptor TLR4 and co-receptor MD-2 (myeloid differentiation factor 2) to detect LPS [6,7]. CD14 binds LPS only in the presence of the soluble lipopolysaccharide-binding protein (LBP). TLR4 is a membrane spanning protein that can form a heterodimer with co-receptor MD-2 and together recognize a common pattern on LPS. TLR is associated with a family of inborn immunity receptors that present an extracellular sphere of leucine-bountiful repeats, a single trans-membrane portion, and a small cytoplasmic signaling area that involves the adapter protein MyD88. In addition, enzymes involved in the biosynthesis pathway for Lipid A are attractive targets for the development of new antibiotics [8]. While LPS is deemed as a principal ligand, CD14 can validate other pathogen-linked molecules such as lipoteichoic acid. Thus, the structures of these proteins constitute how our immune system differentiates LPS molecules from architecturally alike host molecules and enhances our intuition for developing new anti-sepsis drugs. As a vital part of the outer membrane of Gram-negative bacteria, LPS provides architectural integrity to the bacteria and increases the negative charge of the cell membrane to fortify the whole membrane structure. Interestingly, if LPS is mutated or removed many Gram-negative bacteria expire; nevertheless, LPS is not required to a few Gram-negative bacteria such as *Neisseria meningitides*, *Moraxella catarrhalis*, and *Acinetobacter baumannii* [9]. In the case of non-pathogenic features of bacterial ecology such as surface adhesion, bacteriophage sensitivity, and interactions with predator amoebae, LPS is also connected.

LPS is comprised of three structural regions: lipid A, core oligosaccharide, and O-antigen. Amongst these three regions, the lipid A region is the active part and under normal conditions consists of a polyacylated β(1–6) linked glucosamine disaccharide dependent phospholipid acting as a hydrophobic anchor for LPS, and is responsible for the toxic behavior [3,10]. The KDO (3-deoxy-d-manno-oct-2-ulosonic acid) patch exists in the structure. The smallest active parts of LPS are commonly known as Re-LPS. The core diglucosamine structure is phosphorylated and furnished with several fatty acids that are harbored in the outer leaflet of the bacterial cell membrane [11]. Lipopolysaccharide is characterized as having molecular sequences to which innate immune system reacts. A strong immune response is initiated that gives a premonitory signal to the body of bacterial infection [12].

The carbohydrate region of LPS can be precisely recognized with the help of carbohydrate biosensors. When it comes to biosensors based on nanomaterials, Au-NPs (nanoparticles), carbon materials, magnetic nanoparticles (MNPs), and quantum dots (QDs) are popular in the fabrication of carbohydrate biosensors. Metallic nanoparticles (NPs) have largely been implemented to facilitate conjugation with biomolecules for the improvement of all types of biosensors. To date, biomolecule affinity based metal NPs are the basis for the best acceptable assays due to simple separation by centrifugation and also due to their quick assembly with thiolated recognition molecules. As a consequence of extensively using metal NPs, remarkable advances have been achieved for enhancing bio-probe methods in the fields of localized surface-plasmon resonance (LSPR), electrochemical methods, and surface-enhanced Raman scattering (SERS). So, this approach has gained significant recognition because of being amenable, cost-effective, and of considerable simplicity and sensitivity. The unique properties of nanomaterials including their compelling catalytic activities and greater surface area, along with outstanding photonic and electronic hallmarks, makes them desirable to integrate with different functions to bring about a wide range of biological applications and help in selective detection of carbohydrates [13]. Over the last few decades, development of new approaches for the sensitive, selective and rapid detection of LPS has been increasing with great interest, as they are common contagious agents found in humans, animals and plants. Intensifying the monitoring of bacterial LPS is essential in food control, industry, research, and biodefense. New methods have been reported for whole cell bacteria detection [14] and methods are also needed for LPS detection. This review will provide an outline of recent approaches in the application of nanomaterials for the construction of biosensors in detecting lipopolysaccharide (LPS) species. We also discuss structural properties, functionalities and application of nanomaterial dependent lipopolysaccharide (LPS) biosensors.

## 2. Structure of Lipopolysaccharide

LPS is a macromolecular glycolipid of molecular mass 10–20 kDa composed of three structural units:

(1) A long as well as bifurcated hydrophobic lipid A region attached to the carbohydrate chain mainly accountable for toxic nature of the molecule;
(2) A hydrophilic core polysaccharide chain associated with immunogenicity;
(3) A periodic hydrophilic O-antigenic oligosaccharide side chain specific to the bacterial serotype [15].

The lipid A region composed of the phosphorylated diglucosamine backbone is mainly responsible for the myriad in vivo and in vitro activities of endotoxins. Lipid A is the main structural component of Gram-negative bacteria that stimulates the innate immune system [16]. Endotoxins have good heat stability, i.e., boiling for 30 min does not destabilize endotoxin and they are resistant to oxidizing agents. Lipid A has a phosphorylated N-acetyl glucosamine dimer having six to seven fatty acids attached, but in general six fatty acids are found and all of them are saturated. Some of them are attached to N-acetyl glucosamine dimer while others are esterified to the three fatty acids. Lipid A chain has enormous architectural diversity when seen in different bacterial species. Variation can be in terms of the number and length of acyl chains and also there may be other groups substituting at the positions of phosphate moieties [12,17].

R-polysaccharide or core (R) antigen is linked with six positions with one NAG (N-acetylglucosamine), whereas the R antigen incorporates a chain of sugars: KDO-Hep-Hep-Glu-Gal-Glu-GluNAc. Heptose and 2-keto-3-deoxyoctonoic acid (KDO) are two abnormal sugars in the core polysaccharide. KDO is distinctive and consistently found in LPS and it has been used as a benchmark in tools for LPS detection. Core polysaccharide binds O-polysaccharide or somatic (O) antigen made up of continual oligosaccharide subunits of 3–5 sugars, where individual chains vary their length to at most 40 repeating units. Generally, core polysaccharide is smaller than O-polysaccharide and O-polysaccharide comprises the hydrophilic domain of the lipopolysaccharide molecule. The significant antibody-binding portion or antigenic determinant of the Gram-negative cell wall is in the O-polysaccharide. Antimicrobial peptides such as cecropins possess a wider spectrum of activity against Gram-positive and Gram-negative bacteria. The maximum sugar segment varies between species of Gram-negative bacteria in the O-side chain. A minimum of 20 various sugars commonly appear and many of those are unique in characteristic dideoxyhexoses, naturally existing in Gram-negative cell walls. O-polysaccharide provides different types of antigenic *Salmonella* as well as *E. coli* and major other strains of Gram-negative species due to the variation in sugar content. R-strains (rough strains) are assembled because of the nonappearance of the O-region, on the other hand smooth strains or simply S-strain are generated by the availability of O-portion, besides the terminal part exhibits immunological recognition of the O-antigen [15]. The bacteria which produces smooth LPS (S-LPS) possesses inner core (Lipid A proximal) and outer core oligosaccharides. Lipooligosaccharides (LOSs) are produced in bacteria which lack O-polysaccharides. Hydrophilic disaccharide region and hydrophobic acyl chain region are both prone to variations when it comes to Lipid A modification. Each bacterial species have their own number and type of alterations in the structure and in the mode of regulation. Some Gram-negative bacteria such as in *Pseudomona aeruginosa*, *Rhizobium etli* and *Aquifex pyrofilus* show unusual lipid A structures in terms of acyl chain length, distribution, absence of phosphate moieties, presence of galacturonic acid residues, and some modifications in glucosamine units. The altered lipid structure might be helpful to the bacteria for thermal tolerance and in undergoing symbiotic association with plants. During the course of time, bacteria have evolved and modified their LPS structure in order to survive in harsh conditions. LPS is a dynamic molecule in bacterial cell surface that is being remodeled with various enzymes present in the bacteria by complex mechanisms [8,18].

## 3. Fate of LPS in the Human Body

Our innate immune system has great potential to recognize an array of microbes and the major elements of their cell membrane including sugars, lipids, and proteins. The toll-like receptor (TLR) family plays key roles in detecting microbes and producing inflammatory responses [19]. Bacterial lipopolysaccharide when recognized by the innate immune system can lead to uncontrollable cytokine production which in response to it results in cardiovascular collapse and hemodynamic instability which causes fatal sepsis syndrome. As soon as LPS is released in our body it encounters serum protein LBP (LPS binding protein). Recognition of Gram-negative bacteria by the complement system leads to opsonization and lysis. Opsonized bacteria are recognized by phagocytes (monocytes, macrophages, and polymorphonuclear leukocytes). The binding to LBP rapidly catalyzes the transfer of LPS to m-CD14 (membrane-bound CD14) or s-CD14 (soluble CD14) as confirmed by various biophysical techniques such as FRAP (fluorescence recovery after photobleaching) [20]. CD14 being a GPI-anchored protein (glycosyl phosphatidylinositol) lacks transmembrane and intracellular domains. Therefore, additional transmembrane receptors work in coordination with LPS-CD14 complex to bring about LPS-induced cellular activation. Molecular events that lead to signal transduction have been explored to understand the exact mechanism of signal transduction. LPS is bound initially with CD14 was then transferred to the TLR4-MD-2 complex. FRET (fluorescence resonance energy transfer) which is another biophysical technique revealed that LPS was also associated with a heterogeneous complex of four receptors comprising of heat shock proteins Hsp70, Hsp90, chemokine receptor 4 (CXCR4), and growth differentiation factor 5 (GDF5) in the lipid bilayer. Triggering receptor expressed on myeloid cells (TREM)-1 has been shown to be involved in inflammatory responses by bacteria on monocytes and neutrophils. Different cell types have their own way of recognizing LPS. It was suggested that LPS cellular activation occurs in the plasma membrane by lateral diffusion of LPS molecules with transmembrane proteins which assist and initiate signaling by steric stress. Different supramolecular arrangements are involved to explain how the conserved individual molecules of CD14 and TLR4 have managed to deal with the rapidly changing pathogenic world [21].

LPS when released during bacterial cell division or death plays a crucial role in the pathophysiology of inflammation induced septic shock. When LPS enters the blood stream, the response by the monocytes and phagocytic cells is the release of cytokines such as tumor necrosis factor-α (TNF-α), interleukin-6 (IL-6), and interleukin-8 (IL-8). If the cytokines are overexpressed this may lead to multiple organ damage. Sepsis is thought to be a most common fatal disease promoting mortality rates in intensive care units (ICU), even though there is no essential and secure drug treatment identified as of yet. Several precarious effects of antibiotics accelerate the release kinetics of LPS by bacterial death so that it can stimulate the immune system to secrete cytokines and thus initiate endotoxin shock reaction. In the condition named sepsis or septic shock, immune cells are activated on encountering bacteria or bacterial components and produces enormous variety of inflammatory mediators such as cytokines, chemokines, and prostaglandins, along with lipid mediators, and reactive oxygen species. These compounds induce vasodilation, myocardial suppression, and interact directly with the coagulation system. The resulting intravascular coagulation causes hypoperfusion and hypoxia leading to organ failure. This is often the initial lethal stage of sepsis where the patient experience multiple-organ failure, involving the lungs, kidney, and liver. In addition, hypoperfusion may impair the gut-mucosal barrier which may allow bacteria to enter the lymph nodes, several organs, and the circulation and worsen the condition [22]. There is evidence that Kuppfer cells in the liver are important for efforts to clear and detoxify LPS [23]. LPS taken up by Kuppfer cells is released to liver parenchymal cells and possible enzymatic removal of fatty acids occurs and digestion of O-antigen. There is a very serious need for developing anti-inflammatory treatments that can have both antibacterial as well as be neutralizing against LPS characteristic effects [24]. 

An important and complex question has been whether the monomeric or an aggregated (multimeric) form of LPS is the active form for stimulating cytokine production. LPS is an amphiphilic structure and above a critical concentration will form aggregates such as micelles or other structures. In serum, LPS can bind to other proteins including lipoproteins and it has been reported that LPS activity is neutralized on binding to high density lipoprotein (HDL) [25]. It has been reported that the monomeric form of LPS from a strain of *E. coli* was active at stimulating a murine pre-B cell line [26]. The physical behavior of Kdo_2_-Lipid A, a chemically pure form of LPS, was examined and it was found to undergo a gel to liquid crystalline phase change at 36.4 °C [27]. The concentration above which the compound no longer existed as monomers solely was found to very significantly with temperature, being 0.3 nM at 37 °C and 3 nM at 25 °C. These data were interpreted in light of a report comparing the effect of LPS on CD14^+/+^ macrophages with that of CD14^−/−^ macrophages in which a higher concentration of LPS was required to stimulate production of TNFα by CD14^−/−^ macrophages [28]. It was concluded that there is an m-CD14 dependent pathway responding to monomeric LPS and an m-CD14 independent pathway responding to LPS aggregates. In a related study, the multimeric form of a synthetic LPS from *E. coli* was found to be the active form in terms of stimulating human monocytes [29]. It was reported that larger size LPS aggregates were more rapidly internalized by human monocytic THP-1 cells and murine macrophages, but without change in the stimulatory effects [30].

## 4. Methods of LPS Detection

Diverse detection methods are used to in order to detect the endotoxins. These methods include the rabbit pyrogen test, the monocyte activation test, and the *Limulus*-based test methods. The rabbit pyrogen test was the first method for detection of LPS, approved by US Food and Drug Administration. In this test, sterile test substances are injected intravenously into rabbits and the consequence is the rise in rabbit body temperature which is measured. There are tests which are simple, highly sensitive and specific to endotoxin detection such as LAL (Limulus Amoebocyte lysate) which is used quite often. In 1956, Bang discovered that amoebocytes from horseshoe crab agglomerate upon reacting with endotoxin due to a protease cascade. The LAL is based on a coagulation cascade triggered by endotoxins and involves three serine protease zymogens [31]. Factor C is autocatalytically activated by LPS to an active form, which then activates Factor B to an active form which then converts the pro-clotting enzyme to the clotting enzyme. The resulting enzyme cleaves two peptide bonds in coagulogen to yield a gel called coagulin [32].

Bang and Levin devised a method for LPS detection using lysates of amoebocytes, hence the name LAL was given to the assay. Some improved methods (chromogenic, turbidimetric, or viscometric) have been introduced to enhance the LAL test. However, several commercial techniques have been developed with quantity and quality serving for rapid and cost-effective LPS monitoring assays. In addition, since LAL is dependent on an enzymatic reaction, yields are dependent on the activity of protease. Furthermore, it has been reported that LAL has proven sensitivity to a few polymeric types of glucose. LPS is the pyrogen of major concern due to its high stability and pyrogenicity [33]. A comparison of the rabbit pyrogen test with the LAL assay revealed that while results from the two different tests correlated well, the LAL assay was more sensitive (detection limit of 0.06 EU mL^−1^ versus >5 EU mL^−1^ kg body weight^−1^ for the rabbit pyrogen test). In these comparisons, control standard endotoxin from *Escherichia coli* strain O55:B5 was added into hepatitis B vaccine preparations. The notation EU refers to ‘endotoxin unit’ and is a measure of activity rather than of mass. Activity of an endotoxin can vary with structure, aggregation state, and other factors. Using a reference endotoxin, the conversion of 1 EU equals approximately 0.1 ng is recommended [34]. The rabbit pyrogen test took 5 h to complete, while a LAL assay by visual clot observation required one hour for incubation [35]. Kinetic turbidimetric LAL assay and kinetic chromogenic LAL assay were reported as having sensitivities of 0.015 EU mL^−1^ and 0.005 EU mL^−1^, respectively. A modern chromogenic LAL assay in the form of a kit of reagents suitable for use with a microplate reader requires 10 min for incubation of sample and LAL lysate followed by 6 min for reaction with the chromogenic substrate Ac-Ile-Glu-Ala-Arg-pNA which releases p-nitroaniline for detection at 405–410 nm [36]. A standard curve must also be prepared in advance, and a linear response is claimed over the range 0.1–1.0 EU mL^−1^. A wide range of sensitivities for the LAL test has been compiled [37], depending on the sample source, bacterial species and strain, and detection method, with values from 0.005 to 50 EU mL^−1^ (0.0005–5.0 ng mL^−1^) typical and possible sensitivity down to 0.001 EU mL^−1^. Recently, an LPS assay based on recombinant Factor C has been introduced [38], which removes the need for harvesting of amoebocytes from horseshoe crabs and is thus animal free. In this assay, LPS activates Factor C which then cleaves a fluorogenic substrate. The commercial assay kits based on recombinant Factor C are useful over the same range as the LAL assay. The activity of LPS from *Klebsiella pneumoniae*, *Serratia marcescens*, *Bordetella pertussis* and *Pseudomona aeruginosa* was found to vary significantly using a protocol using a fluorescence microplate reader and a recombinant Factor C assay [38].

It is also reported that the effectiveness of the current testing procedures is under debate with respect to a recently reported phenomenon of low endotoxin recovery (LER) also known as ‘masking of naturally occurring endotoxin’, defined as inability over time to recover more than 50% of the activity. Previous reports have shown that endotoxin spiked in a matrix containing a chelator and a surfactant cannot be recovered by dilution or magnesium replacement. LER and the associated masking effects have been confirmed. Depending on the source, preparation and degree of purification, the LPS can possess different structure in terms of lipid A acylation, substitution and distribution of sugar residues, composition, and endotoxin activity [39]. The effect of LPS aggregation on the response of the LAL assay and the extent of LER has been noted and a mechanism proposed for LER [40]. In this mechanism, LPS aggregates are viewed as being in a cubic arrangement stabilized by Ca^2+^ ions and addition of chelator and surfactant results in removal of the Ca^2+^ ions and leads to replacement of LPS by the surfactant on the surface of the aggregates. While the LER mechanism requires further study, it appears that addition of chelator and surfactant causes a change in aggregation that causes a reduction in potency. The LER phenomena make assays for LPS based on its activity subject to the LER issue. As it is known that LPS aggregation depends on temperature and salt concentration, assays in general must be done under carefully controlled conditions.

The word “sensor” is derived from the Latin word “sentire” which means ‘to identify’. A sensor is composed of a receptor and a transducer. The role of the receptor is to receive the chemical/physical stimulus while transducer converts the signal in a form that can be analyzed. Magnetic sensors, thermometric sensors, optical sensors, radiation detecting sensors, electrical, and electrochemical are some of the different classes of sensors worked upon so far. Amongst these, biosensors incorporate physical and chemical sensing techniques. Biosensors have organic/biological recognition elements on their surface that help in selective uptake of analytes from the medium, making them quite distinct from others [23]. The term “biosensor” was coined by Cammann, as analytical devices that must be highly specific and remain unaffected by physical parameters such as pH, temperature, etc. Fabrication of biosensors able to detect LPS incorporates knowledge from chemistry, biology, and engineering [41]. The difference between the two is that LPS is the free form while endotoxin refers to LPS attached on the bacterial surface. LPS is released from the bacteria when it undergoes cell lysis and division or during its growth [42]. Different types of biosensors to detect these have been developed which include.

### 4.1. Protein-Based Biosensors

Highly sensitive and accurate detection of endotoxins is necessary for the development of biopharmaceutical products targeted at Gram-negative bacteria. The interaction of the antimicrobial LPS binding protein CAP18 with lipid A was shown by circular dichroism measurements to occur through cationic and hydrophobic patches on the protein [22]. Both hydrophobic interactions and electrostatic interactions can contribute to the binding affinity of LPS. Lipopolysaccharide-binding protein (LBP) is a lipid-transfer protein that may expedite monomeric LPS molecules movement from the aggregation of LPS to CD14; a hydrophobic attachment island shapes the receptor complex with the help of CD14 [43]. CD14 conveys monomeric LPS to TLR4 and to myeloid differentiation protein-2 (MD-2), resulting in the necessary reordering of TLR4 and contributing to the secretion of pro-inflammatory cytokines after the fusion of LPS and TLR4/MD-2 complexes [44]. Comparable to bacterial/permeability-increasing protein (BPI) which comes from the lipopolysaccharide-binding protein (LBP) lineage and can potentially restrict LPS activation at some concentrations, serum amyloid p elements and cationic protein (CAP18) are also described to have incorporation and detoxification abilities against LPS at local areas of inflammation [45,46,47]. These last three proteins are familiar as endotoxin-neutralizing proteins (ENPs) and are synthesized from amoebocytes of horseshoe crab such as *Limulus* anti-LPS factory [48]. Protein-based sensors generally cross-bind to other cellular components; therefore, there is a need to develop sensors of high sensitivity and specificity [33]. Enhanced green fluorescent protein (EGFP) has been used as a fluorescent biosensor for bacterial endotoxin. The bioactive component of LPS (lipid A), is involved in interaction with these sensors. In this method, computational techniques were used to design mutants of enhanced green fluorescent protein bearing one or two endotoxin binding domains. Detection of lipid A from *E. coli* lysates was performed by fluorescence quenching (excitation wavelength = 488 nm, detection wavelength = 508 nm) with a dissociation constant of 8.15 × 10^−8^ M achieved for the mutant bearing two recognition domains [49]. This same protein was capable of detecting LPS from *E. coli* O55:B5 to a sensitivity of 5 ng mL^−1^.

The limulus amebocyte lysate (LAL) assay reagents were used in the combination with a quartz crystal microbalance to detect endotoxin on the basis of the decrease in oscillator frequency due to the viscosity increase that occurred due to the ultimate effect of endotoxin triggered conversion of coagulagen to coagulin by the activated clotting enzyme. Detection of endotoxin spiked into human plasma samples was successful over a linear range from 0.005 to 10 EU mL^−1^ [32]. Quartz tuning forks have also been used to detect the coagulation of LAL assay reagents induced by endotoxin from *E. coli* O157:H19 or the presence of the intact bacteria. The coagulation caused a decrease in the oscillation amplitude and a detection range of 0.001–5 EU mL^−1^ was found for the endotoxin and of 10^2^–10^7^ CFU mL^−1^ for the intact bacteria [50].

### 4.2. Peptide-Based Biosensors

**Figure 2:**
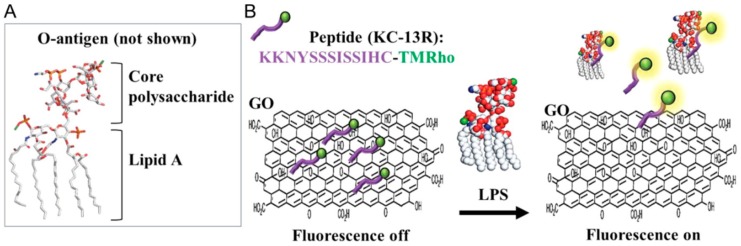
(**A**) Representation of LPS structure. (**B**) Fluorescently labeled peptide coupled with graphene oxide acting as a biosensor for LPS by showing variation in intensity of fluorescence upon its interaction with LPS. (Reproduced with permission from reference [52]), copyright ACS).

The peptide polymyxin B (PMB) is an amphipathic cationic polypeptide that blocks the effects of LPS by binding to the negatively charged LPS leading to the death of Gram-negative bacteria by destabilizing their outer membrane. Antimicrobial peptides (AMPs) are another class of recognition molecules which have been associated with the chemistry of the immune system. They are present in the host’s innate immune system and serve as part of the defense machinery against microbial invasion. Unlike antibody-based detection techniques, AMPs are highly stable under extreme environmental conditions and bind semi-selectively to microbial cell surfaces causing membrane disruption. Magainin I falls under the class of AMPs which are linear amphipathic peptides, being unstructured in solution phase but becoming helical upon interaction with target membranes. For the first step, magainin I as a capture molecule was immobilized by the conjugation of biotin-avidin on silanized glass slides and direct covalent binding to examine molecules and positive control antibodies over sensor substrates across the cross-linking agent. The avidin–biotin chemistry has demonstrated that this interaction is very useful for a strong stable sensor. Depending on both specific and non-specific affinity as well as variability, detection limits for *E. coli* and *Salmonella typhimurium* onto covalently immobilized magainin I of 1.6 × 10^5^ and 6.5 × 10^4^ cells mL^−1^ were found. When magainin I was immobilized indirectly using biotin modification at the C-terminus and binding to a layer of avidin, the detection limits were not as good being 6.8 × 10^5^ and 5.6 × 10^5^ cells mL^−1^, respectively. The detection limits for *E. coli* are higher than for the similar antibody-dependent probes with the advantage of improved stability. It is suspected that there may be other AMPs with greater binding affinities. Direct binding of magainin I reduced non-specific cell attachment but at the same time enhanced detection limits for both *Salmonella* and *E. coli*. [51]. Use of a dispersion of graphene oxide (22 μg mL^−1^) modified by tetramethylrhodamine labeled synthetic LPS binding peptide (KC-13, sequence: KKNYSSSISSIHC) was used for fluorescence based detection of LPS from four common bacterial pathogens (*Escherichia coli*, *Klebsiella pneumonia*, *Samonella thyphosa*, and *Pseudomonas aeruginosa*) [52]. Binding of LPS to the labeled KC-13 peptide released the LPS–KC13 complex from the graphene oxide and resulted in an increase in fluorescence (excitation wavelength = 480 nm, detection wavelength = 572 nm). A detection limit of 130 pM was achieved, noted as the lowest to date for a synthetic LPS sensor based system. The authors note that the molar mass of commercial LPS varies from 4 to 20 kDa and that they are assuming a molar mass of 10 kDa. The molar mass of LPS is not a well-defined number due to heterogeneity and aggregation; hence values in units of molarity cannot be reliably converted to other units if authors do not provide information on molar mass at least as an approximation. The representation of this approach is shown in Figure 2.

### 4.3. Aptamer Based Biosensors

Aptamers are single-stranded oligonucleotides that possess high stability, reversible denaturation, specificity and high affinity for the target molecule. A gold electrode has been modified with 3-mercaptopropionic acid (MPA) to immobilize single stranded DNA (ssDNA) aptamers as probes that are LPS specific. It was observed that the amine terminated aptamer exhibited high affinity for LPS (from *Escherichia coli* 055:B5 (L4524)) of K_d_ = 11.9 nM. In this report, cyclic voltammetry (CV), as well as electrochemical impendence spectroscopy (EIS) were used for the characterizing each step of the gold surface modification. Change in charge-transfer resistance (∆R_ct_) versus the logarithmic value of LPS concentration exhibited a wider dynamic monitoring range of 0.001–1 ng mL^−1^ and a linear relationship. Selectivity for LPS was high in presence of pDNA, RNA, and bovine serum albumin (BSA). The sensor could be regenerated at low pH for re-use. During the last two decades, aptamers have been used to target various substrates with high specificity and affinity, thus making them promising probes for the fabrication of aptamer-based biosensors. In contrast to protein-based sensor probes such as antibodies or enzymes, DNA aptamers are highly stable, and can be synthesized with high reproducibility and purity [53]. To overcome the limitations of small surface-to-volume ratio and prolonged incubation time to study kinetics, an aptamer that employs magnetic beads (MB) has been developed with high sensitivity, quicker detection and less sample consumption. An aptamer-based sandwich assay with magnetic beads and two endotoxin binding aptamers was explored. Endotoxin-conjugated sandwich complex gave green fluorescence which was quantified using flow cytometry. The carboxylic groups on the magnetic beads link to aptamer Apt B2 via amide linkages. The other aptamer Apt B9 was labeled with FAM (carboxyfluorescein), which formed an endotoxin-B9 complex in solution. This complex specifically interacts with MB-B2 and produced a sandwich complex. The magnetic aptasensor so formed was able to detect LPS (from *Escherichia coli* O55:B5 (L4524)) within the range of 10^−8^ to 10^0^ mg mL^−1^ including in the presence of interferents that coexist with the endotoxin, such as RNA, proteins, glucose, sucrose, and bovine serum albumin (BSA) [54].

### 4.4. Antibody-Based Biosensors

Antigen-antibody binding has been used to detect microbes and various species including endotoxins, viruses, spores, and bacteria. For instance, a gold electrode was fabricated with an insulating layer of 2 µm of thermal oxide and functionalized with heterobifunctional cross-linkers to immobilize polyclonal antibodies for sensitive bacterial detection. The biosensor was evaluated for *E. coli* O157:H7 and the bacteria became attached to the immobilized antibodies [55]. A biosensor has been developed for the detection of *Salmonella typhimurium* by immobilizing a polyclonal antibody on the surface of a magnetostrictive platform which is made up of amorphous ferromagnetic alloys [56]. The additional mass due to bacteria binding causes a shift in the resonance frequency, and for a sensor of dimensions 2.0 mm × 0.4 mm × 0.15 μm a detection limit of 5000 CFU mL^−1^ was obtained. Examination of the sensor surfaces by SEM showed good correlation of bacterial counts with resonance frequency shifts. *E. coli* O157:H7 is a highly aggressive form that has the capability to produce toxins resulting in urinary tract infection (UTI), common in women. It may cause kidney failure if not detected properly in time and may lead to hemolytic uremic syndrome (HUS), resulting in death. Biological sensing and binding of bacteria has been performed using Anti-*E. coli* LPS specific antibody covalently bound to gold nanowire arrays (GNWA) prepared using an alumina template to produce 10 nm dimension standing gold nanowires after template removal. Dithiobissuccinimidylundecanoate was used to modify the GNWA and then covalently conjugated to the LPS specific antibody [57]. A linear response between the capacitance and the logarithm of the cell count was observed and a detection limit of 10 cells in a 0.173 cm^2^ area was demonstrated.

### 4.5. Cell-Based Biosensors

Macrophage cell based sensors have been in use to detect LPS (from *Escherichia coli* O157:H7) based on shifts seen in the infrared spectroscopy spectrum for the amide I vibration. Silica oxide substrate was selected for patterning gold electrode microarrays and was further covered with fibronectin to immobilize macrophage cells; 0.1 µg mL^−1^ LPS was sensed using cell-based sensors on gold microarray electrodes. The LPS toxin may induce cell death by bringing out morphological changes in cells such as cell rounding and membrane blebbing [22,55]. Surface plasmon resonance studies of human embryonic kidney cells (HEK-293) plated onto glass/gold/poly-l-lysine surfaces exposed to LPS from *E. coli* showed reproducible decreases in reflectance brought on by LPS induced changes in cell morphology in the form of collapse of intracellular structures [58].

In vitro tests for pyrogens based on monocyte activation have been proposed based on the sensitivity of monocytes for pyrogens. In this technically sophisticated technique, the products are incubated with peripheral blood mononuclear cell fraction (PBMNC) and cell conditioned media assayed for pyrogenic cytokines. Various cell lines have been used to carry out pyrogen tests in vitro including MONO MAC 6 and THP-1 cell lines. The readouts include TNF-α, IL-1ß, IL-6, and neopterin. However, IL-6 is considered better as it is released entirely and can be estimated completely [59].

## 5. Biosensors Based on Nanomaterials

Bioreceptors are biological species and include antibodies, proteins, oligonucleotides, cells or whole organisms that are capable of selective recognition in a complex sampling medium. It has been observed that selectivity can be enhanced when nanomaterials are used together with bioreceptors. Up to now, a large number of nanomaterials including noble metal nanoparticles, quantum dots, carbon nanomaterials as well as metal oxide nanoparticles have been actively explored for the sensitive and selective detection of pathogenic bacteria. LPS can bind to all kinds of surfaces including surface functionalized nanoparticles depending on the ability of nanoparticles to establish Coulombic and van der Waals interactions with the endotoxin molecules. It has been reported that the hydrophobic part (Lipid A) binds to the surface of nanoparticles. Additionally, LPS binding to nanomaterial depends on the surface coating and surface charge (relevant in physiological media). The major contributor to nanoparticle-LPS interaction is electrostatic interactions. Negatively charged polysaccharide portions of LPS have strong affinity for positively charged nanoparticles surfaces [60,61].

In recent years, advances have been made in the field of nanomaterial modified sensing electrodes for rapid electron transfer and rapid response. For the rapid and sensitive detection of microbial toxins, nanomaterials are considered ideal due to their unique chemical and physical properties. Nanomaterials when coupled with biomolecules can selectively bind to the glycans of pathogenic bacteria. Different morphologies and shapes of nanomaterials have been developed in recent years to be used for sensing such as nanoribbons, nanowires, nanotubes, etc. Pathogen detection using nanomaterial based sensors works by binding with the carbohydrate structures on bacterial membranes. This interaction generates a signal transduction for the monitoring of the pathogen. It is still a challenge to develop low cost rapid-detection biosensors with improved selectivity and sensitivity. Improved performance is seen when nanomaterials with the size scale of 10^−9^ m have been used as biosensors and due to their high surface area, the binding ability with the target analyte is enhanced. Intrinsic properties of nanomaterials make them suitable for their use as biosensors. Nanomaterials including metal nanoparticles (mNPs), magnetic nanoparticles (MNPs), quantum dots (QDs), upconversion nanoparticles (UCNPs), carbon nanotubes (CNTs), graphene, carbon nanodots (CDs), and silica nanoparticles (SiNPs) can be used for biosensing applications [48,53]. Hybrid nanomaterials combine the properties of two materials and give synergistic effects which include surface-enhanced Raman scattering (SERS), fluorescence resonance energy transfer (FRET), enhanced catalytic performance, magneto-optical effects, and improved mechanical properties. To work as signal transducers, the above listed properties play a crucial role. Interaction between the target pathogen and the nanomaterial brings about a change in the properties of nanomaterial used for sensing that could be sensed via electroanalytical techniques. Therefore, nanomaterial-based biosensors exhibit good potential to be used in detection systems for pathogens and endotoxins [62]. The choice of nanomaterial is very crucial when it comes to use in a biosensor. Noble metals, quantum dots, magnetic nanoparticles, and dye-doped nanoparticles are currently of major interest as biosensor substrates. Different nanomaterials are employed based on different types of signal transduction mechanisms. Different mechanisms include optical signal transduction, magnetic methods and electrochemical methods. Novel structures with defined control over optical, magnetic, and electrochemical properties are developed. The most commonly used optical techniques for detecting endotoxin are fluorescence and surface plasmon enabled spectroscopies. Quantum dots (QDs) are widely used semiconductor nanocrystals where modifying the particle size and chemical composition can alter the emission wavelength. Dye-doped silica nanoparticles are less prone to agglomeration and have high luminescent intensity. Furthermore, the silica matrix protects the luminophores from oxidation thereby stabilizing the fluorescent signal. Gold and silver nanoparticles have stable localized surface plasmon resonances (LSPR) within the nanoparticles and therefore, are widely used in biosensors relying on optical signal transduction. Noble metals are photostable optical labels due to their intrinsic feature of having large molar extinction coefficients [63].

### 5.1. Gold Nanoparticle-Based Biosensors

**Figure 3:**
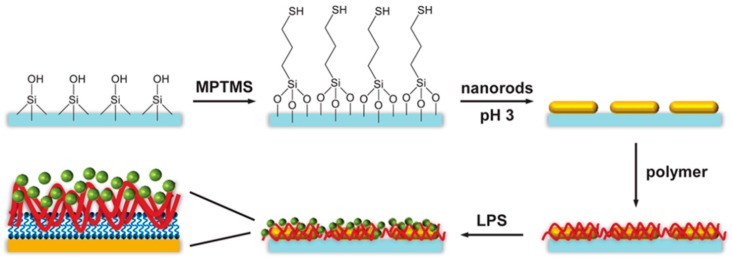
Gold nanorods immobilized on functionalized glass substrates for sensing experiments for LPS. (Reproduced with permission from reference [64], copyright ACS).

Gold nanoparticles hold a special place in the applications such as biomolecule sensing, photothermal therapy, bioimaging, and drug delivery. They are a promising candidate for sensing due to excellent biocompatibility, good stability, and shape dependent unique optoelectronic properties. Gold nanorods are a potential tool to detect pathogenic bacteria, as shown in Figure 3. Modifying the nanorod surfaces with different polymers affected binding of LPS thereby altering the interactions of various types of LPS with nanoparticles [64]. The gold nanorods of dimensions 42.3 ± 5.6 nm length and 11.6 ± 1.6 nm width were immobilized onto glass slides treated with mercaptopropyltrimethoxysilane and the coated with various polymers including sodium polyacrylate, poly(allylamine) hydrochloride, heparin, polydiallyldimethyl ammonium chloride, and methoxyl polyethylene glycol thiol. The red shift in the longitudinal plasmon resonance of the gold nanorods from 783 nm was used to measure the binding of LPS extracted from *Pseudomonas aeruginosa*, *Salmonella enterica*, and *E. coli*. The number of LPS molecules bound per nanorod was determined from the refractive index shift as determined from the shift in the plasmon absorbance wavelength and ranged from 0 to 400 per nanorod. The strongest binding was seen for LPS from *E. coli* to the poly(allylamine hydrochloride) coated nanorods, measured as an association constant of 1.1 ± 0.3 × 10^10^ M^−1^.

**Figure 4:**
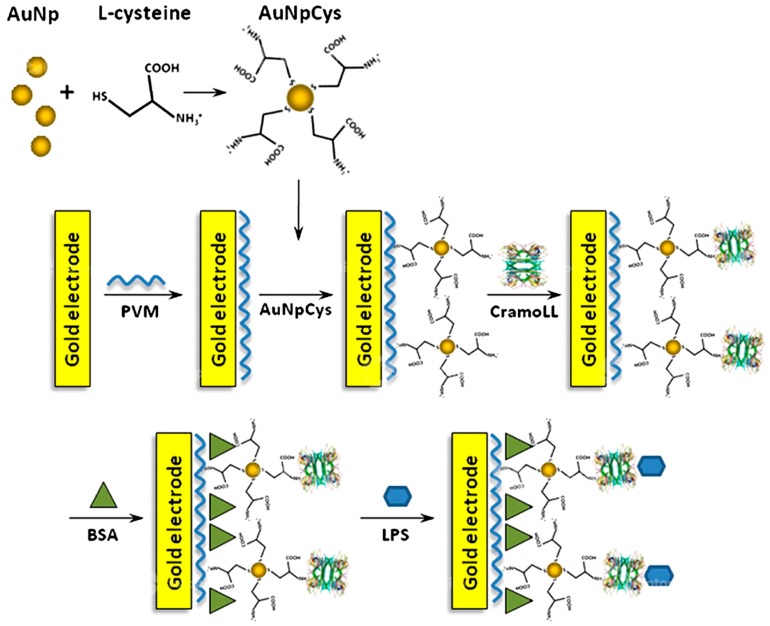
Schematic representation of the PVM-AuNpCys-CramolL-BSA-LPS biosensor system. (Reproduced with permission from reference [65]). PVM: poly(vinyl chloride-co-vinyl acetate-co-maleic acid); BSA: bovine serum albumin.

Electrochemical methods employing gold and silver nanoparticles include quantification of changes seen in metal conductivity with interaction of nanoparticles surface with the analyte [63]. Label-free detection of bacterial LPS has been done involving the use of lectins which are carbohydrate specific proteins. Different bacteria can be recognized if the sugar residues find a suitable match from the available lectins and the binding will be specific for the two and can be very well exploited. A sensitive electrochemical biosensor based on impedance spectroscopy has been made using a new lectin named CramoLL isolated from *Cratyliamollis* seeds and can recognize Gly/Man and glycoproteins. CramoLL can bind to monosaccharides such as methyl-α-d-mannoside (MeαMan) by forming hydrogen bonds. MeαMan interacts either by direct hydrogen-bonding or through hydrophobic contacts with the protein residues. Gold nanoparticles coated with cysteine acting as the substrate electrode with further modification using poly(vinyl chloride-co-vinyl acetate-co-maleic acid) (PVM) has been developed. PVM shows good adhesion to the electrode and the negative charge over its surface electrostatically attracts CramoLL lectin. In addition, gold nanoparticles have high surface-to-volume ratio and high surface energy to provide a stable immobilization of biomolecules. Fast and direct electron transfer between electrode material and electroactive species was possible by using gold nanoparticles. LPS was seen to adsorb over the surface of PVM-AuNpCys-CramoLL-BSA system in the form of aggregates with height of about 34 nm determined from atomic force microscopy (AFM) image analysis. The binding of CramoLL to bacterial LPS is based on protein–carbohydrate interaction similar to that seen in ConA. Glycosyl and mannosyl residues on the surface of LPS are responsible for recognizing CramoLL [65]. In these experiments, LPS from *S. enterica* serotype *typhimurium* (strain ATCC7823), *K. pneumonia* (strain ATCC15380), *S. marcescens* (strain ATCC21639) and *E. coli* (strain ATCC13027) were used. The response, measured as shift in charge transfer resistance, was greater for LPS from *S. marcescens* and *E. coli* LPS than for that from *S. enterica* and *K. pneumoniae*, attributed to differences in structure of the LPS oligosaccharides. Figure 4 depicts the mechanism of surface modification of the LPS biosensor created by the above mentioned technique.

A colorimetric assay based on the electrostatic interaction of gold nanoparticles with LPS aggregates in solution was reported [66]. The LPS (from *Escherichia coli* 055:B5) aggregates as bilayers in solution above a very low concentration. LPS is negatively charged due to the two 2-keto-3-deoxyoctonate units and the phosphate groups of Lipid A. Gold nanoparticles will be attracted to these bilayers and will associate bringing the nanoparticles closer together and resulting in a population of aggregated gold nanoparticles red-shifted in the absorbance spectrum. It was found that (A_650_/A_525_)^1/2^ shifted linearly with LPS concentration. Detection of LPS from *E. coli* O55:B5 was achieved over a range from 5 to 90 nM and with a detection limit of 0.33 nM (3.3 ng mL^−1^). The molar mass of the LPS was determined to be 10 kDa by electrophoresis. Nanomaterial–biological interfaces are complicated and widely studied due their potential in in vivo biological studies. Nanoparticles interact with the bacterial cell wall but are not internalized as for mammalian cells. The outer membrane of Gram negative bacteria is mainly composed of LPS and therefore, the nanoparticles interact with the lipid A region of LPS to become anchored onto the surface. Localized surface plasmon resonance (LSPR) refractometric sensing using gold nanorods have been used to monitor the interaction between biomolecules and metal nanoparticles by measuring the change in refractive index due to target binding. Surface charge and surface chemistry are critical for the understanding of gold nanorod–LPS interactions [64]. LPS structure plays an important role in deciding the extent and location of binding.

### 5.2. Silver-Based Biosensors

Stabilized silver nanocolumns have been used for the detection of endotoxin. SAMs (self-assembled monolayers) of MPA and EDC-NHS (1-ethyl-3-(3-dimethylaminopropyl)carbodiimide and N-hydroxysuccinimide) were formed on the surface in order to stabilize the silver nanoparticles and activate the carboxylic acid groups on the SAM. Limit of detection (LOD) of the biosensor was measured to be 340 pg mL^−1^ for *E. coli*. Silver nanocolumns were deposited on glass substrates using the thermal evaporation method. Polymyxin B, a polycationic peptide, was immobilized on the surface of the nanoparticles to interact with the endotoxin. Refractive index sensitivity was measured using the LSPR biosensing technique. This biosensor was able to detect endotoxins selectively when present with other biological species [67]. 4-mercaptophenylboronic acid (MPBA) functionalized silver nanoparticles (AgNPs) have been used utilized for a novel colorimetric detection of Gram-negative bacteria [68]. The location of bacteria was traced with the help of the reaction of MPBA with cis-diol groups in the saccharides present on bacterial cell membranes. The agglomeration of MPBA-AgNPs was inhibited by bacterial cells giving rise to a color change. These nanoparticles attach to the bacterial surface keeping them separated resulting in the color change from yellow to brownish red. This is due to the formation of a six membered planar boroxine ring through self-dehydration condensation of boronic acid group. The limit of detection was 0.9 × 10^4^ CFU mL^−1^ and the response time was 20 min. Combination of nanomaterials with electrochemical techniques is indeed an efficient strategy for pathogen detection. Bacterial antibody immobilized on the surface of silver nanoflowers via covalent conjugation has been used for the rapid detection of *E. coli* O157:H7. Bovine serum albumin (BSA) conjugated 3D Ag nanoflowers proved to be a powerful material with good conductivity and biocompatibility for pathogen detection [69]. This biosensor was capable of detecting *E. coli* strains over a range of 3.0 × 10^2^–3.0 × 10^8^ CFU mL^−1^. It was also proposed that this biosensor might be used to detect disease related proteins, cancer cells, and heavy metal ions.

### 5.3. Silica Nanoparticle-Based Biosensors

Porous silicon (or nanoscale silicon) has many advantages making it a suitable candidate to be used for biosensing applications. Its large surface area provides a platform for the attachment of various species of interest. Furthermore, the nanomaterial has room temperature luminescence visible by eye thereby making it an effective transducer. An organic receptor, ter-tryptophan ter-cyclopentane (TWTCP) that binds specifically to lipid A has been used to modify the porous silicon surface for functionalizing it to detect endotoxin [70]. More recently, triple dye doped fluorescent silica nanoparticles have been used to detect multiple bacteria. An effective method based on biotin-labeled aptamer and streptavidin-conjugated silica fluorescent nanoprobes was developed for the detection of bacteria *S. typhimurium* showing high affinity to its membrane proteins [71]. Dye doped silica nanoparticles possess several advantages which includes signal amplification, photostability, and surface modification for biological applications [72]. Silica and silica-based nanoparticles have gained significant interest in the recent years to be used as nanoprobes. The surface of silica nanoparticles can be modified with different ligands and biomolecules such as proteins, oligonucleotides, and others for biological applications. The nanoparticles when conjugated with antibody or aptamer can selectively detect bacterial cells. The dye doped silica-based nanoparticles have been used for immuno-labeling of bacteria *E. coli* O157:H7. High intensity fluorescent dye-doped nanoparticles were combined with antibodies to form probes that could identify *E. coli* O157:H7 [73].

### 5.4. Magnetic Nanoparticles in Biosensing

**Figure 5:**
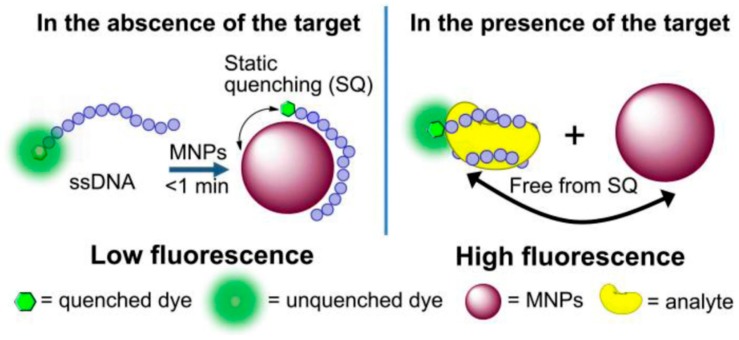
Schematic diagram showing dye labeled DNA aptamer with magnetic nanoparticles (MNPs) and the change in signal on encountering target molecule (Reproduced with permission from reference [80]).

Superparamagnetic nanoparticles are widely used in biosensor applications due to their capability to magnetize under an applied magnetic field. Recently, the use of superparamagnetic nanoparticles for biosensing has become popular. A magnetic immunoassay gave good results in the detection of LPS from *Francisella tularensis* and concentrations of less than 0.1 ng mL^−1^ were detected [74]. Biosensing using the amalgamation of magnetic nanoparticles has been used to highly effective. A magnetic glyconanoparticle (MGNP)-based system has been reported to detect *E. coli* within 5 min and also remove 88% of the target bacteria from the medium. The MGNP system was able to distinguish three strains of *E. coli* (ORN 178, ORN 208, and ES) based on their response pattern. It is beneficial to use magnetic nanoparticles for pathogen biosensing due to the high surface/volume ratio that helps in binding carbohydrates and thereby capturing pathogens. Magnet mediated separation is possible as the size of nanoparticles (NPs) is smaller than bacteria leading to attachment of multiple NPs on the bacterial cell walls. Additionally, small NPs can bring about faster detection due to their speedy binding kinetics in solution. Detection of *E. coli* strain ORN178 was possible when incubated with silica-coated magnetite NPs with D-mannose attached through an amide linkage. Using magnetic separation and staining with fluorescent dye PicoGreen, *E. coli* has been reliably detected with a limit of 10^4^ cells mL^−1^ [75]. Magnetic nanosensors can bind specifically to the bacterial target with high sensitivity without any interference caused by other bacteria. Functionalized superparamagnetic iron oxide nanoparticles have been used to detect *Mycobacterium avium* spp. *Paratuberculosis* (MAP), through magnetic relaxation [76]. Surface functionalized magnetic nanoparticles selectively bind to specific molecular targets. Nanoscale magnetic materials possess strong magnetic properties and can be used as biosensing labels. Upon varying their composition, size and morphology, we can modulate their magnetic properties to suit the biosensing application. Three different types of biosensors that make use of magnetic nanoparticles labels are (a) magnetic relaxation switches, (b) magnetic particle relaxation sensors, and (c) magnetoresistive sensors [77]. A novel approach has been developed to separate endotoxins and bacteria from the blood stream by the use of magnetic nanoparticles modified with the synthetic ligand zinc-coordinated bis(dipicolylamine) (bis-Zn-DPA). The synthetic ligand bis-Zn-DPA forms a coordination complex with specific lipids making it selective for the rapid separation of both Gram-negative and Gram-positive bacteria and their endotoxins. The rate of binding of the ligand with the bacteria was faster compared to antibody-based approaches, shortening the incubation time prior to separation. The approach used here completely removed bacteria at a higher flow rate than the previously reported methods. Bis-Zn-DPA was immobilized on the surface of amine terminated Fe_3_O_4_ nanoparticles via carbodiimide chemistry. The binding of bis-Zn-DPA was found to be highly specific with *E. coli* as seen from the microscopic images where the bacteria is stained green with fluorescein (FITC)-labeled ligand [78]. For sensitive pathogenic bacteria, multifunctional graphene magnetic nanosheets decorated with chitosan (GMCS) have been utilized as a promising biosensor. Surface capping with chitosan enhanced the biocompatibility, mechanical strength and tensile strength. Graphene has great potential in biosensing for detecting single bacterium. Moreover, the graphene sheet increases the stability of magnetic nanoparticles, Fe_3_O_4_ due to its large surface area. Graphene-nanomagnetic hybrid materials have been seen to achieve rapid, sensitive and low cost detection of pathogenic bacteria such as *Pseudomonas aeruginosa* and *Staphylococcus aureus* [79]. For expanding the applications of magnetic nanoparticles in biosensing, the combination of DNA and magnetic nanoparticles (MNPs) has been carried out in recent years. Fluorescent dye labeled DNA was reacted with polyethylenimine (PEI) coated magnetic nanoparticles to capture the fluorescence signal when the system is utilized for biosensing. Figure 5 shows that the interaction of dye labeled ssDNA with MNPs results in quenching of fluorescence while in the presence of target molecules the formation of DNA-MNPs complex is interrupted leading to restoration in fluorescence signal. Highly sensitive and selective detection of LPS have been made using this sensor with limit of detection (LOD) ~35 ng mL^−1^ and a linear range from 50 ng mL^−1^ to 10 µg mL^−1^. The PEI-MNPs based sensor was found to be better than a DNA-GO based system as it decreases the background noise by eliminating non-specific interactions [80].

### 5.5. Carbon Nanomaterials as Biosensors

For the use of biosensors, carbon nanotubes (CNTs) and graphene have been considered as promising materials in the scientific research community due to their unique chemical and physical properties [81]. Their nanoscale dimensions and graphitic surface chemistry make them suitable for their use as biosensors for microbial toxins [82]. A fluorescent turn-on sensor for the detection of lipopolysaccharide (LPS) using peptide assembled graphene oxide (GO) has been developed. LPS purified from *E. coli* O111:B4, *Pseudomonas aeruginosa*, *Salmonella typhosa*, and *Klebsiella pneumonia* were used. LPS from *P. aeruginosa* gave a higher response that the other three LPS sources. The fluorescence modulation when dye labeled peptide interact with the GO was monitored. The detection limit was found to be 130 pM [52]. Combination of graphene nanocomposites with antibodies and/or aptamers have been employed to fabricate electrochemical biosensors for specifically and selectively detecting endotoxins released by bacterial cells. Graphene nanocomposites have been used as efficient labels for generating electrochemical signals. Reduced graphene oxide-doped polypyrrole/pyrrole propylic acid nanocomposite has been used to fabricate immunosensors. Graphene oxide nanoplatelets (GONPs) were directly used as electroactive labels for aptasensing of mycotoxin. Hypersensitive detection of LPS has been achieved using a three-way DNA hybridization process with the combination of electroactive toluidine blue–graphene–gold nanoparticles nanocomposites. The limit of detection for this material was found to be 8.7 fg mL^−1^. These biosensors exhibit outstanding performance in detecting microbial toxins [83].

### 5.6. Quantum Dots (QDs)-Based Biosensors

Quantum dots have been employed to be used as biosensors due to their similar size as that of biomolecules. Photoluminescence (PL) is modified when QDs interact with biomolecules. Chitosan modified CdS quantum dots (CdS@CTS) have been used as biosensors due to their affinity for bacterial membranes. It has proved to be an ultrafast, sensitive, direct, and biocompatible material for sensing LPS. *Staphylococcus aureus* and *Pseudomonas aeruginosa* were detected at low concentrations of 150 and 200 CFU mL^−1^, respectively, in a short span of time [84]. Quantum dots are ideal for use as optical biosensors due to their unique property of quantum confinement effect. QDs possess several unique properties such as large absorption coefficients, size tunable light emission, superior signal brightness, resistance to photobleaching, and excitation of multiple fluorescence colors concomitantly. CdTe QDs have been used to detect LPS of *S. marcescens*. The nanomaterial was made selective by surface modification with thioglycolic acid (TGA) along with lectin Con A [85]. A sensitive and quick bacterial screening system has been developed utilizing CdSe/ZnS QDs functionalized with colistin for the detection of *E. coli*. Semiconductor nanocrystals can serve well as luminescent sensors for the detection of bacterial cells. Development of QD-based sensors for biological systems is a growing field providing a rapid, simple, and sensitive approach. Common fluorophores such as GFP and luciferase have drawbacks that include low signal-to-noise ratio and fast photobleaching and therefore QDs are being used for which the fluorescence emission wavelength is a function of particle size. The presence of ZnS in the outer shell helps to enhance photostability in the quantum dot structure that makes it superior to traditional organic dyes used so far as fluorescent probes. The interaction of QDs and target bacteria is based on antigen-antibody interaction. Colistin (polymyxin E) shows prominent activity against Gram-negative bacteria by interacting with the LPS in the outer membrane of the bacteria through ion displacement thus, destabilizing the cell membrane. Colistin binds specifically to the lipid A portion [86].

### 5.7. Upconversion Nanoparticles as Biosensors

Over the past decade, rare earth metal doped upconversion nanomaterials have been synthesized for use in biosensing. Upconversion nanoparticles have the ability to emit visible light under near infrared radiation which leads to less scattering and more penetration making them suitable for in vitro and in vivo uses. In comparison to organic dyes and QDs, UCNPs have an advantage in having high quantum yields, high photostability, and low background signals. An IR laser can excite multiple analytes from different UCNs. In recent years, UCNs have been used for the sensitive detection of oligonucleotides [87]. A specific multiplex method for the detection of three bacteria namely, *Staphylococcus aureus*, *Vibrio parahemolyticus*, and *Salmonella typhimurium* simultaneously has been reported making use of multicolor upconversion nanoparticles (UCNPs) as labels in combination with aptamers that serve the function of recognizing specific sites on the pathogenic entity. UCNPs were made by doping with rare-earth ions such as Er^3+^, Tm^3+^, and Ho^3+^. The limits of detection for this method were 25, 10, and 15 CFU mL^−1^ for *S. aureus*, *V. parahemolyticus*, and *S. typhimurium*, respectively. UCNPs are preferred over other fluorescent biolabels due to their unique optical and chemical features and lack of autofluorescence [88]. Interesting results were seen when UNCPs based FRET aptasensor was used to detect pathogenic *E. coli* ATCC 8739. The observed detection range was from 5 to 10^6^ CFU mL^−1^ with a detection limit of 3 CFU mL^−1^ with biosensing done within 20 min. By modifying the aptamer sequences this methodology can be applied to detect a broader range of targets. This is an ultrasensitive, rapid, and highly specific FRET based bacteria detection method. Herein, gold nanoparticles were in conjugation with the bacterial target aptamer and on the other hand UCNPs act as donor with aptamer functionalization of complimentary DNA sequence. FRET was observed due to the spectral overlap of complimentary DNA sequences leading to fluorescence quenching between AuNPs and UCNPs. When pathogenic bacteria are in the vicinity, aptamers bind leaving behind UCNPs giving rise to fluorescence [89].

### 5.8. Other LPS Biosensing Systems

Devices for bacterial diagnosis in the form of sensors which has the ability to detect different forms of LPS have been looked for in recent times. Functionalized liposomes fall under this category. Liposomes synthesized from 1,3-diacetylenic lipids have found great application in the field of biosensors. LPS recognition via these sensors is based on the principle of electronic tongue. In this technique, analyte is exposed to multiple receptors which interact with the given analyte differently giving rise to a diagnostic pattern, or fingerprint for that specific analyte. The fingerprints generated can be used to detect LPS in different strains of Gram-negative bacteria. Tryptophan and tyrosine residues are used to bind with carbohydrates and therefore, the signaling component of the sensor that is polydiacetylene is modified using these protein residues in order to interact well with the LPS. Polymerized liposomes synthesized using above mentioned technique are sensitive to LPS which was seen through colorimetric analysis. Colorimetric response (CR) was quantified by measuring the absorbances in the presence and absence of LPS. The sensor response was also seen in the presence of additives which tend to aggregate LPS by interacting with the phosphate groups in it. Applying this strategy, a diagnostic fingerprint for *E. coli* O26:B6 was successfully developed. The CR values varied from 0 to 55% depending on the bacterial strain and experimental conditions. This was a novel approach for fabricating sensors for specific strains of bacteria [90]. Recently, a fluorescent turn-on sensor for detecting micromolar levels of bacterial LPS has been developed. Polydiacetylene liposomes surface modified with fabricated pentalysine peptide and histidine in the ratio 1:9 respectively have effectively shown fluorescence signal only in the presence of LPS. Significant increase in the fluorescence signal was observed at E. coli concentrations as low as 4.91 × 10^8^ CFU mL^−1^ with liposomes at a concentration of 40.0 µM. The sensor worked on the principle of FRET. The two interacting moeities were pentalysine oligopeptide connected to tricosadiynoic acid with napthalic acid fluorophore and histidine connected to tricosadiynoic acid. The mixture of two gave no signal as fluorescence is quenched due to energy transfer from napthalic acid fluorophore to cross-linked polymer. Binding LPS restored the fluorescence, present even at sub-micromolar concentrations. The response observed was found to be highly selective for LPS. The binding constant found from Stern–Volmer analysis of increased signal was found to be 1.5 × 10^6^ M^−1^ [91].

Structure-activity correlation between polymyxin B (PMB) and synthetic cyclic peptide analogs of PMB using surface plasmon resonance (SPR) has been used to determine the kinetics of association and dissociation of the synthesized peptides with endotoxin. The results from the study prominently showed the vital role played by amphiphilicity of the peptides and hydrophobic forces in the interaction between PMB analogs and endotoxin. The cyclic hepta- and deca-peptides were synthesized using a solid phase peptide synthesizer employing Fmoc and Opfp chemistry. The peptides were covalently immobilized on the sensor chip at concentrations of 40 µg mL^−1^ in 10 mM sodium acetate, pH 4.8, using amine coupling kit. The measurements were carried out in 10 mM HEPES, pH 7.4, 150 mM NaCl, 3.4 mM EDTA. SPR relies on the mass change so the interaction can be studied without the use of labels. SPR experiments report a total binding event. The binding constant K_a_ for cyclic decapeptide, polymyxin nonapeptide (PMBN), and cyclic heptapeptide were found to be 3.5 × 10^5^, 2.1 × 10^5^, and 2 × 10^3^ M^−1^ respectively [92].

Information on the LPS sensing strategies reported in this section is summarized in Table 1. There are clearly some rapid methods reported with detection times of under 10 min. Techniques based on the optical response of nanoparticles in solution are especially attractive given the possibility for simpler measurements using standard laboratory equipment. There is a clinical need to measure relatively low levels of LPS, as the median concentration of LPS in patients with sepsis has been reported as 0.3 ng mL^−1^ [93]. There is a need for more studies applying detection methods for LPS in human serum or similar environments. There is also a need for improved detection limits together with a broader linear range. The quartz crystal microbalance method appears especially sensitive amongst the reported results using non-electrochemical methods.

## 6. Detection of LPS via Electrochemical Sensing Techniques

Electrochemical biosensing for LPS detection requires enzyme modification with a biological recognition element such as an enzyme, antibody, molecular receptor, microorganism, or nucleic acid aptamer. The presence and concentration of LPS can be detected using electrochemical biosensors based on LPS binding interactions and electrochemical transduction. Sensors prepared electrochemically can be highly effective in detecting LPS in trace amounts. The carbohydrate portion of LPS interacts with divalent cations such as Cd^2+^, Cu^2+^, Pb^2+^, Zn^2+^, and this property has been exploited to develop sensors using metal complexes and regeneration of the LPS sensor [94]. A gold electrode surface was modified by mercaptopropionic acid that was then activated with EDC/NHS and reacted to conjugate nitrilotriacetic acid (NTA). The complex of NTA and Cu^2+^ was found to bind to the carbohydrates of LPS. Detection using electrochemical impedance spectroscopy gave a linear relation between charge transfer resistance and LPS concentration over the range 0.0001–0.1 ng mL^−1^. Removal of the Cu^2+^ using EDTA could be applied to regenerate the sensor.

A novel electrochemical technique for lipopolysaccharide (LPS) detection has been reported wherein a derivative of ferrocenylboronic acid is used in combination with an enzyme-modified electrode. In this study, a gold electrode was modified by casting bovine serum albumin (BSA) and diaphorase together with glutaraldehyde to form a cross-linked membrane. Ferrocenylmethyl dimethylamine was added together with diaphorase cofactor NADH. In the presence of LPS, the current due to redox cycling of the ferrocene was reduced by the association of the LPS glycosyl units with the boronic acid group, which resulted in the ferrocenylboronic acid derivative becoming less able to penetrate the membrane. The modified electrode showed high selectivity and rapid response of 5 min with a detection limit for LPS extracted from *E. coli* O127:B8 of 50 ng mL^−1^ [95].

A new strategy has been worked upon for designing an electrochemical LPS sensor which is based on the human recombinant toll-like receptor 4 (rhTLR4) and myeloid differentiation-2 (MD-2) complex [96]. In this strategy, gold electrodes were surface modified with dithiobis(succinimidyl undecanoate) (DSU) to form self-assembled monolayers followed by blocking with BSA. The complex rhTLR4/MD-2 binds specifically with endotoxin and was successfully immobilized on the electrodes by covalent conjugation to an activated form of the self-assembled monolayers. Cyclic voltammetry (CV) and differential pulse voltammetry (DPV) were used to observe the decrease in current for the Fe(CN)_6_^3−/4−^ redox probe resulting from the interaction of the complex with endotoxin from *E. coli* O55:B5. A linear response was seen between 0.0005–5 EU mL^−1^ (EU = endotoxin units) and the sensor was selective against a phospholipid.

An approach to LPS detection using aptamers attached to a thin layer of gold nanoparticles was found to be successful for detecting LPS from *E. coli* O55:B5 [97]. A thin layer of Au nanoparticles was formed by electrodeposition from 10 mM HAuCl_4_ for 20 s at −0.2 V (vs. Ag/AgCl) yielding oblate particles of 200 nm average diameter. A thiolated aptamer specific for LPS was immobilized and electrochemical impedance spectroscopy applied for endotoxin detection, achieving a linear range of 0.1–10.24 ng mL^−1^. A set of 10 LPS aptamers were developed using the SELEX approach and found to have dissociation constants K_d_ in the nM range [98]. Electrochemical impedance spectroscopy was applied to gold electrodes modified with the best of the thiolated aptamers and a linear response to LPS was found over the range of 0.01–1.0 ng mL^−1^. The test took only 15 min compared to 1.5–2.0 h for the traditional LAL assay. Gold nanoparticle aggregates with the particles bridged and decorated by Cu^2+^ ions were used for detection of LPS using differential pulse voltammetry [99]. In this study, an aptamer for LPS was conjugated to a gold electrode surface through mercaptopropionic acid. In solution, gold nanoparticles (17–20 nm) modified with cysteine were linked into 400 nm aggregates by Cu^2+^. Binding of LPS to the aptamer on the electrode surface followed by exposure to the Cu^2+^ linked aggregates would result in interaction of Cu^2+^ with bound LPS and bring the aggregates to the surface where the Cu^2+^ could be detected using stripping voltammetry by DPV. Two linear ranges were observed for peak current vs. LPS concentration, one from 0.05–1.0 pg mL^−1^ and a second from 1.0–10 pg mL^−1^. The affinity of LPS for Cu^2+^ was found to be greater that for Zn^2+^, Ni^2+^ or Cu^2+^. Aptamers to LPS were immobilized on gold electrodes modified with Au atomic clusters (0.5–2.0 nm in size) and the response was measured by the decrease in the current due to reduction of gold upon LPS binding [100]. A linear range from 0.01 aM–1.0 pM was found using differential pulse voltammetry and also with electrochemical impedance spectroscopy. Due to the heterogeneity and aggregation of LPS, the interpretation of molarity concentrations for LPS is unclear.

A nanocarbon surface formed by unbalanced magnetron sputtering was subsequently fluorinated using electron cyclotron resonance sputtering to yield a surface onto which poly-ε-lysine was cast as a thin film cross-linked with BSA [101]. The fluorinated surface had the unusual property of efficient inner sphere electron transfer for ferrocene but blocking electron transfer of dissolved Fe^2+/3+^. It was found that the film had affinity for LPS, to which a ferrocene modified polymyxin B (FcPMB) could bind. The bound FcPMB amplified the current from dissolved Fe^2+^ and this effect could be used for LPS detection, achieving a linear range of 0.02–200 ng mL^−1^. Polymyxin B can be conjugated to gold electrodes modified with 4,4′-dithiodibutyric acid using EDC and sulfo-NHS chemistry [102]. The affinity of polymyxin B for LPS could be detected using electrochemical impedance spectroscopy, and a range of response from 0.2–0.8 ng mL^−1^ was found from the shift of the charge transfer resistance.

A highly selective scheme for the detection of LPS using the catalytic properties of CeO_2_ metal oxide frameworks was developed [103]. In this scheme, gold nanoparticles were formed by electrodeposition on a glassy carbon electrode and then modified with a single strand DNA hairpin probe. Application of a solution of LPS together with Zn^2+^ and a double-stranded DNA probe resulted in dissociation of the duplex with one strand binding to LPS and the other to Zn^2+^ to form an active DNAzyme. The DNAzyme cleaved the hairpin probe at the surface, releasing one of the strands. Subsequently, CeO_2_ metal-oxide framework materials that were prepared with surface amine groups and decorated with gold nanoparticles bearing a second hairpin DNA probe were applied. The second hairpin probe hybridized with the remaining strand of the hairpin probe on the surface that had been cleaved by the DNAzyme. Finally, the CeO_2_ materials in the form of rod-like structures bound to the surface were used to oxidize ascorbic acid that was detected by differential pulse voltammetry. The sensing scheme showed very high selectivity, a detection limit of 3.3 fg mL^−1^ and a linear range from 10 fg mL^−1^ to 100 ng mL^−1^.

**Figure 6:**
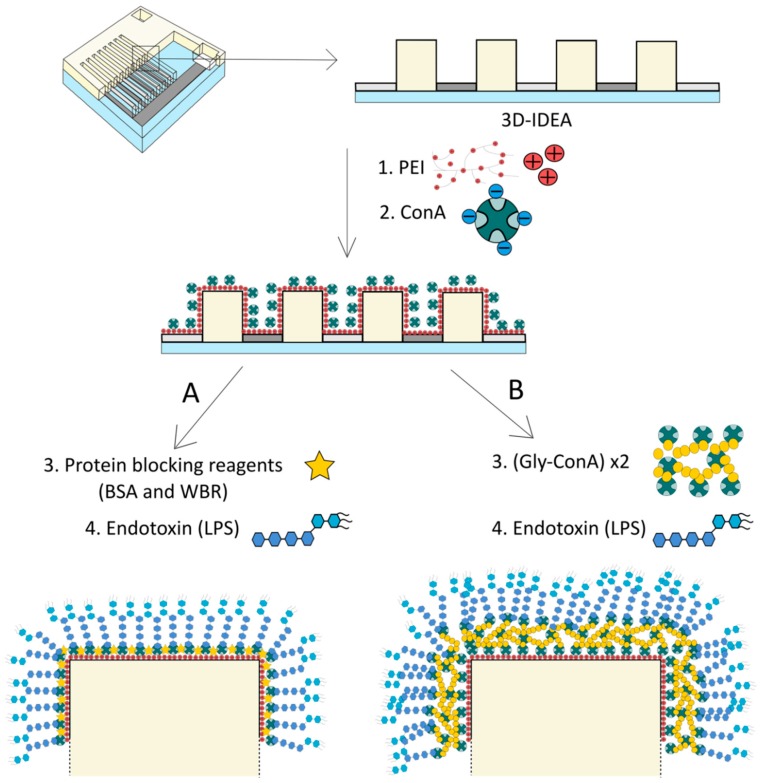
Sensor design and sequential steps to achieve surface biofunctionalization. (Reproduced with permission from reference [104]).

Biosensors are considered as a promising alternative to the classical techniques used so far. For achieving high selectivity the biorecognition element has to be immobilized on the sensor surface. Lectins are one such biorecognition element which is more stable and have higher surface coverage than other biomolecules such as antibodies. Concanavalin A (Con A) is a carbohydrate binding protein that has high affinity towards some bacteria, viruses, cells, and endotoxins. Electrochemical impedance spectroscopy (EIS) is a powerful technique to register surface changes due to recognition events. In this study, a three-dimensional interdigitated electrode array (3-D IDEA) wherein electrode digits are separated by insulating barriers was used as a biosensor transducer with enhanced sensitivity. It is a promising label-free biosensor which is sensitive to changes in the electrical charge distribution. Under applied potential, the main portion of the current reaches close to the surface of barrier and in this way the sensitivity is increased when compared with respect to the planar surfaces. Initial anchoring layer on the sensor surface was created by layer-by-layer (LBL) technique using polyethyleneimine (PEI) forming a multilayer coating of positively charged polycation. Surface modification is essential to increase sensitivity and reduce non-specific interactions as schematically represented in Figure 6. The sensor prepared by this technique can detect bacterial LPS in 20 min with the limit of detection (LOD) of 2 µg mL^−1^ [104].

The results for the electrochemical detection of LPS from the reports summarized here are shown below in Table 2. It can be seen that electrochemical methods can offer rapid measurement times down to as short as 10–20 min, which would be highly attractive in clinical settings and is shorter than the time for the rabbit pyrogen or some implementations of the LAL assay. A drawback of electrochemical approaches for clinical settings would be the sophisticated nature of the instrumentation and data interpretation. Of the methods used, it appears that electrochemical impedance spectroscopy is highly promising, as is the use of aptamers as recognition elements. Aptamers are especially attractive as they are chemically robust. Given that the chromogenic LAL assay on microplates takes about 10 min also, there are multiple options for fairly quick LPS assays. Selectivity will be important and LAL offers selectivity, as do aptamers. It is likely that other recognition strategies such as chemical binding or use of lectins will be less selective. The reporting of detailed data comparing performance of binding of different forms of LPS remains a challenging future question.

## 7. Conclusions

In this review, we have summarized the various techniques used so far as well as the upcoming strategies for the sensitive and highly specific detection of LPS using nanomaterials. We have started with the discussion of lipopolysaccharide structure and function and progressed to discuss the techniques used earlier including rabbit pyrogen test and LAL test with their advantages and some gaps that were filled with further advancements in detection technologies. The significance of conventional biosensors based on proteins, peptides, antibody, aptamer, and cell based have been examined. However, conventional methods are mainly lab-based and require longer times for detection. Finally, we surveyed the use of nanomaterials as biosensors for the sensitive detection of LPS that are able to give reliable, accurate, and fast results. Different nanomaterials along with their sensor performance have been discussed in this review. We further assessed the use of metal nanoparticles (mNPs), magnetic nanoparticles (MNPs), quantum dots (QDs), upconversion nanoparticles (UCNPs), carbon nanotubes (CNTs), graphene, carbon nanodots (CDs), and silica nanoparticles (SiNPs) for biosensing applications. Some of the main advantages and disadvantages of the methods discussed here are summarized in Table 3. In the detection of LPS, one must consider the specificity of the binding interaction used combined with the sensitivity and range of the physical method used for signal detection.

Electrochemical methods, including those based on nanostructured electrode surfaces, can be quite sensitive and impedance spectroscopy appears as an especially promising method. A potential drawback of impedance spectroscopy is that it is a fairly sophisticated experiment to conduct and presents complex data. In terms of nanomaterials, methods that make use of the optical response of modified nanoparticles―either as localized surface plasmon effects for gold nanoparticles, or of fluorescence for quantum dots or carbon nanostructures―appear quite attractive if they can be directly introduced into the sample. The development of a quick and sensitive method using readily available optical readouts applied to LPS containing samples mixed with nanoparticles sensitive to LPS appears within reach. The achievement of a similar goal for detection of whole bacteria also appears within reach. Aptamers appear promising for providing high selectivity and sensitivity to LPS once their development using SELEX has been achieved. Magnetic nanoparticles have the main advantage of being useful for capture and separation of bound bacteria or LPS for further analysis. Other means of binding LPS via electrostatic interactions or to lectins may prove less selective in human plasma or more complex sample matrices. The recent availability of recombinant Factor C from the LAL assay opens additional possibilities for its usage in future assays. There is clearly a need to reduce the need for harvesting LAL from horseshoe crabs. The extracted knowledge of fabrication of these biosensors to meet the detection requirements can provide guidance to build much more advanced probes for LPS sensing.

## Figures and Tables

**Table 1:** Summary of selected reports on the detection of LPS. When available, the LPS source, measurement time, detection limit, and range of detection are shown in the table.

Material	Method	LPS Source	Sensitivity, Time	Range	Reference
LAL assay	various	various	0.005–50.0 EU mL^−1^ (0.0005–5.00 ng mL^−1^)		[35]
Recombinant Factor C and fluorogenic peptide substrate	fluorescence	various	0.005 EU mL^−1^	0.005–50.0 EU mL^−1^	[38]
Enhanced green fluorescent protein mutant	Fluorescence	*E. coli* O55:B5	5 ng mL^−1^		[49]
Limulus amebocyte lysate	Quartz crystal microbalance	Endotoxin standard Spiked in human plasma	0.005 EU mL^−1^ (0.0005 ng mL^−1^), 30 min	0.005–10 EU mL^−1^	[50]
Dye-labeled LPS binding peptide	Fluorescence quenching on graphene oxide	*E. coli* 0111:B4, *Pseudomonas aeruginosa*, *Salmonella typhosa*, and *Klebsiella pneumonia*	130 pM (molar mass of 10 kDa assumed), equivalent to 1.3 ng mL^−1^, 5 min	0–20 nM, equivalent to 0–20 ng mL^−1^	[52]
Aptamer modified magnetic beads	Flow cytometry/scanning confocal laser microscopy	*E. coli* O55:B5	0.01 ng mL^−1^ Less than 1 min	10^−2^–10^−6^ ng mL^−1^	[54]
Polymer modified gold nanorods	Localized surface plasmon resonance	*Salmonella enterica* serotype typhimirium, *Escherichia coli* 0127:B8	K_a_~10^7^–10^10^ M^−1^ for LPS binding to nanorods, depending on polymer	Not reported	[64]
Cysteamine-modified gold nanoparticles	Localized surface plasmon resonance	*E. coli* O55:B5	0.33 nM, molar mass determined as 10 kDa, equivalent to 3.3 ng mL^−1^, 5 min	5–90 nM	[66]
Superparamagnetic nanoparticles/antibodies	Magnetization	*Francisella tularensis*	0.1 ng mL^−1^, 20–60 min	0.1–1000 ng mL^−1^	[74]
Array of silver nanocolumns/polymyxin B peptide	Localized surface plasmon resonance	*E. coli* O111:B4	0.34 ng mL^−1^, 1 h		[67]
Magnetic nanoparticle/dye labeled aptamer	Fluorescence quenching		35 ng mL^−1^, 40 min	50–10,000 ng mL^−1^	[80]
CdTe quantum dots/Con A	Photoluminescence quenching	*S. marcescens* (strain ATCC 21639)		10–90 fg mL^−1^	[85]

**Table 2:** Summary of selected reports on the electrochemical detection of LPS. When available, the LPS source, measurement time, detection limit, and range of detection are given in the table.

Material	Method	LPS Source	Sensitivity, Time	Range	Ref.
Gold electrode/SAM/aptamer	Electrochemical Impedance spectroscopy	*E. coli* O55:B5	0.001 ng mL^−1^	0.001–1.0 ng mL^−1^	[53]
Gold electrode/polymer/Au nanoparticles/CramoLL lectin	Electrochemical Impedance spectroscopy	*S. enterica* serotype *typhimurium* (strain ATCC7823), *K. pneumonia* (strain ATCC15380), *S. marcescens* (strain ATCC21639) and *E. coli* (strain ATCC13027)	Not reported; most sensitive to LPS from *S. marcescens* at 200 μg mL^−1^, 20 min	Not reported	[65]
Gold electrode/nitrilotriacetic acid terminal SAM/Cu^2+^	Electrochemical Impedance spectroscopy	*E. coli* O55:B5	0.0001 ng mL^−1^	0.0001–0.1 ng mL^−1^	[94]
Gold electrode/diaphorase enzyme/ferrocenylboronic acid	Cyclic voltammetry	*E. coli* O127:B8	50 ng mL^−1^, 10 min	Not reported	[95]
Au electrode/SAM/rhTLR4/MD-2 complex	Differential pulse voltammetry	*E. coli* O55:B5	0.0002 EU mL^−1^ (2.0 × 10^−5^ ng mL^−1^), 30 min	0.0005–5 EU mL^−1^	[96]
Au nanoparticle modified electrode/aptamer	Electrochemical Impedance spectroscopy	*E. coli* O55:B5	0.05 ng mL^−1^, 10 min	0.01–10.24 ng mL^−1^	[97]
Aptamer modified Au electrode	Electrochemical Impedance spectroscopy	*E. coli* O55:B5	15 min	0.01–1.0 ng mL^−1^	[98]
Aptamer modified electrode and modified Au nanoparticles	Differential pulse voltammetry	*E. coli* O55:B5	0.033 pg mL^−1^, 64 min	0.05–1.0 pg mL^−1^; 1.0–10 pg mL^−1^	[99]
Au cluster—aptamer modified electrode	Differential pulse voltammetry	Endotoxin standard	7.94 × 10^−21^ M (molar mass not reported)	0.01 aM–1.0 pM	[100]
Fluorinated nanocarbon electrode/poly-ε-lysine	Cyclic voltammetry	Japanese Pharmacopeia reference standard	0.2 ng mL^−1^	0.02–200 ng mL^−1^	[101]
Polymyxin B modified SAM on Au electrode	Electrochemical Impedance spectroscopy	*E. coli* O111:B4	0.2 ng mL^−1^	0.2–0.8 ng mL^−1^	[102]
Ce-based metal organic framework with Au nanoparticles and DNAzyme	Differential Pulse voltammetry	Not given	3.3 fg mL^−1^ 195 min	10 fg mL^−1^–100 ng mL^−1^	[103]
3D-interdigitated electrode array	Electrochemical impedance spectroscopy	*E. coli* O55:B5	2000 ng mL^−1^, 20 min	0–50 μg mL^−1^	[104]

**Table 3:** Summary of some of the advantages and disadvantages of methods described in this review in terms of binding interaction and signal detection for LPS.

Method	Advantages	Disadvantages
**Binding Interaction**		
*Limulus Amoebocyte* lysate	Inexpensive; ease of use	Reliance on horseshoe crabs; possible variation in lysate potency
Rabbit pyrogen test	Ease of use	Use of rabbits, lack of sensitivity, waiting time
Aptamers	Highly specific, high binding affinity, chemically robust	Aptamer availability
Polymyxin	Inexpensive and stable	Nonspecific electrostatic interactions
Lectin-based detection	Targeting of specific carbohydrate structures	Low binding affinity, affinity for other substrates
Toll-like receptor complex-based detection	Highly selective	Stability and proper immobilization of complex
**Signal detection**		
Optical detection (Localized surface plasmon resonance)	Use of simple absorbance spectroscopy for detection	Sensitivity varies with particle geometry; non-specific binding must be prevented
Optical detection (fluorescence)	High sensitivity	Possible quenching effects or fluorophore degradation
Electrochemical impedance spectroscopy	High sensitivity, label-free	Sophisticated instrumentation and data analysis
Mass-sensitive detection (e.g., quartz crystal microbalance)	Label-free	Response to non-specific adsorption and changes in viscoelasticity
Magnetic nanoparticles	Prospect for separation of target from complex media	More suitable for separation than detection
